# Linkage Group Selection: Towards Identifying Genes Controlling Strain Specific Protective Immunity in Malaria

**DOI:** 10.1371/journal.pone.0000857

**Published:** 2007-09-12

**Authors:** Sittiporn Pattaradilokrat, Sandra J. Cheesman, Richard Carter

**Affiliations:** Ashworth Laboratories, Institute of Immunology and Infection Research, School of Biological Sciences, University of Edinburgh, Edinburgh, United Kingdom; North Carolina State University, United States of America

## Abstract

Protective immunity against blood infections of malaria is partly specific to the genotype, or strain, of the parasites. The target antigens of Strain Specific Protective Immunity are expected, therefore, to be antigenically and genetically distinct in different lines of parasite. Here we describe the use of a genetic approach, Linkage Group Selection, to locate the target(s) of Strain Specific Protective Immunity in the rodent malaria parasite *Plasmodium chabaudi chabaudi*. In a previous such analysis using the progeny of a genetic cross between *P. c. chabaudi* lines AS-pyr1 and CB, a location on *P. c. chabaudi* chromosome 8 containing the gene for merozoite surface protein-1, a known candidate antigen for Strain Specific Protective Immunity, was strongly selected. *P. c. chabaudi* apical membrane antigen-1, another candidate for Strain Specific Protective Immunity, could not have been evaluated in this cross as AS-pyr1 and CB are identical within the cell surface domain of this protein. Here we use Linkage Group Selection analysis of Strain Specific Protective Immunity in a cross between *P. c. chabaudi* lines CB-pyr10 and AJ, in which merozoite surface protein-1 and apical membrane antigen-1 are both genetically distinct. In this analysis strain specific immune selection acted strongly on the region of *P. c. chabaudi* chromosome 8 encoding merozoite surface protein-1 and, less strongly, on the *P. c. chabaudi* chromosome 9 region encoding apical membrane antigen-1. The evidence from these two independent studies indicates that Strain Specific Protective Immunity in *P. c. chabaudi* in mice is mainly determined by a narrow region of the *P. c. chabaudi* genome containing the gene for the *P. c. chabaudi* merozoite surface protein-1 protein. Other regions, including that containing the gene for *P. c. chabaudi* apical membrane antigen-1, may be more weakly associated with Strain Specific Protective Immunity in these parasites.

## Introduction

In regions of the world where malaria is endemic, anti-parasitic protective immunity against infection with the pathogenic blood stages of the parasites is acquired gradually after repeated exposure to malaria [1]–[3]. This immunity is non-sterile, leading to a state of premunition in which low parasite densities are maintained in the host without causing disease symptoms. In contrast to the slow acquisition of protective immunity to malaria under natural conditions, such immunity can be achieved relatively quickly in humans under controlled clinical conditions and in laboratory animals after one, or a few, blood stage-induced infections with a single cloned strain (genotype) of malaria parasites followed by drug cure [4]–[10]. However, while this immunity can be very effective in protecting individuals against blood stage malarial infection with parasites of the same genotype, it is often less effective against challenge infection with blood stage parasites of a different genotype. These observations imply the existence of Strain Specific Protective Immunity (SSPI) in malaria. Thus, the slow acquisition of immunity to naturally acquired malarial infection is likely to be at least partly due to the existence of polymorphism in the target antigens of protective immunity against the parasites (i.e. multiple allelic forms of an antigen-coding gene) [11].

Natural populations of human malaria parasites [12]–[16] and laboratory strains of rodent malaria parasites [17], [18] are, indeed, genetically highly polymorphic. Molecular characterisation of genes for protein antigens expressed on the surface of merozoites during blood stage malarial infection has revealed extensive sequence polymorphism in many of these antigen-coding genes. Prominent amongst them are genes encoding the merozoite surface protein-1 (MSP-1) [19], [20] and the apical membrane antigen-1 (AMA-1) [21], [22]. Both MSP-1 and AMA-1 have been implicated as targets of SSPI in *Plasmodium falciparum* malaria in humans [23]–[28] and in *Plasmodium chabaudi* malaria in rodents [29]–[31].

In a previous study using Linkage Group Selection (LGS) [9] to search for genes encoding targets of SSPI in *P. chabaudi chabaudi*, we located a region of the genome on *P. c. chabaudi* chromosome 8 which contains the gene for MSP-1 [9] as encoding a major target of SSPI. However, the two *P. c. chabaudi* strains, AS-pyr1 and CB, used in that study are genetically identical for the cell-surface, ectodomain region of the gene for AMA-1 (Cheesman and Carter, unpublished data), the other prominent candidate for SSPI in rodent malaria [31]. It is highly unlikely, therefore, that this LGS analysis could have detected AMA-1 as a target of SSPI [9]. In the present study, we have conducted LGS analysis of SSPI on two *P. c. chabaudi* strains, AJ and CB-pyr10, which are genetically different for the genes for both MSP-1 and AMA-1 (Cheesman and Carter, unpublished data). The present analysis has again identified the region on *P. c. chabaudi* chromosome 8 containing the gene for MSP-1 to be under strong SSPI selection. A weaker SSPI response was also found in the region on *P. c. chabaudi* chromosome 9 containing the gene for AMA-1. However, no region in the genome of *P. c. chabaudi* was identified under strength of SSPI selection comparable to that on the region containing the gene for MSP-1. The combined results from these two studies indicate, therefore, that the main force of SSPI is determined by the single, narrow region of the *P. c. chabaudi* genome containing the gene for the protein antigen MSP-1.

## Results

### Characterisation of Strain Specific Protective Immunity in mice for *Plasmodium chabaudi chabaudi* cloned strains CB and AJ

**Figure 1:**
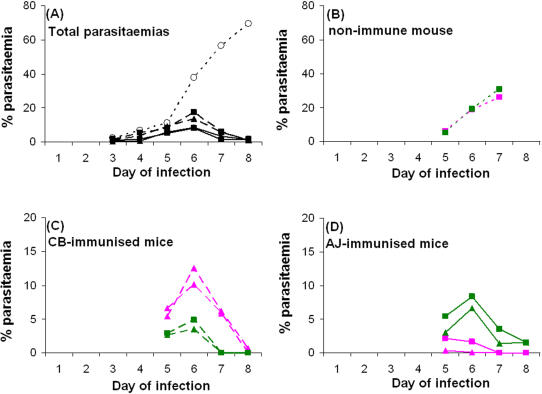
Mixed strain infections of *Plasmodium chabaudi chabaudi* CB and AJ in mice pre-immunised with either strain, or in a non-immune batch mate. (A) shows total parasitaemias during the course of mixed strain infections in immunised and non-immune mice as measured by microscopic examination of thin blood smears stained with Giemsa's stain: non-immune mouse (dotted line with open circles), two CB-immunised mice (dashed lines with filled symbols), two AJ-immunised mice (unbroken lines with filled symbols). Strain specific parasitaemias (AJ in pink; CB in green) are shown (B) in the non-immune mouse, (C) in the two CB-immunised mice and (D) in the two AJ-immunised mice. The strain specific parasitaemias in the mixed strain infections were calculated from the total parasitaemias measured on thin blood smears stained with Giemsa's stain and from the proportions of CB and AJ parasites in the mixtures as determined using strain specific RTQ-PCR (see text). Squares and triangles represent mouse 1 and 2, respectively, in the CB and AJ immunised mice in Fig. 1A, C and D.

Immunity against the blood stages of *P. c. chabaudi* was induced in groups of female CBA mice, five to six weeks old on first infection, by two successive single strain blood stage-induced infections of either *P. c. chabaudi* CB or AJ, drug cured with mefloquine of, as described in *Materials and Methods*. Sixteen weeks after the last mefloquine dose, two CB-immunised mice and two AJ-immunised mice were challenged with a mixture of an equal proportion of blood stage parasites of CB and AJ. A non-immune mouse, which was a batch mate of the immunised mice, was inoculated with the same mixture of blood stage parasites of CB and AJ. Total parasitaemias were measured daily by microscopic analysis of thin blood smears (Figure 1A). The proportions of parasites carrying the CB or AJ alleles of *P. c. chabaudi msp*-1 were determined using strain specific Real Time Quantitative-Polymerase Chain Reaction (RTQ-PCR) analysis of DNA samples obtained from the mixed strain infections between 5 and 8 days after inoculation (see *Materials and Methods*).

In mice previously immunised with either CB or AJ, the total parasitaemias following challenge with the mixed strain infections of *P. c. chabaudi* CB and AJ were greatly reduced relative to the total parasitaemia in a non-immune batch mate infected with the same mixture (Figure 1A). It is clear, therefore, that a strain-transcending immunity must have been present in the CB and AJ immunised mice as both reduced the absolute parasitaemias to a similar degree. Parasitaemias of the strain homologous to the immunising one were, however, consistently lower, and the parasites were eliminated more rapidly, than those of the heterologous strain in the immunised mice (Figure 1C and 1D). By contrast, CB and AJ were present in almost equal proportions throughout the period of observation in the non-immune mouse (days 5 to 8) (Figure 1B).

The differential effect in the strain specifically immunised mice was greatest on day 7 of infection when parasites of the homologous strain were undetectable (<1% parasitaemia) by strain specific RTQ-PCR (see *Materials and Methods*), while parasites of the heterologous strain survived at parasitaemias of ∼6% and 1–3% in CB- and AJ-immunised mice, respectively (Figure 1C and 1D). Single strain (CB or AJ) immunised batch mates of the mice tested in this experiment were used to apply SSPI selection pressure against the uncloned progeny of a genetic cross between CB and AJ, as described in the following section.

### Strain Specific Protective Immune Selection of the Uncloned Cross Progeny between *P. c. chabaudi* Cloned Strains CB and AJ

**Figure 2:**
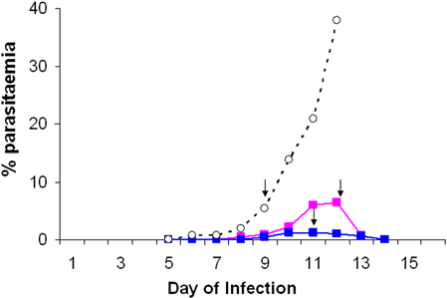
Course of blood stage induced infection of the uncloned CB x AJ cross progeny grown in a non-immune mouse (dotted line with open circles), in a CB-immunised mouse (pink line filled squares) and in an AJ-immunised mouse (blue line with filled symbols). Arrows indicate day of infection when the uncloned cross progeny grown in the immunised mice or the non-immune mouse were sub-inoculated for expansion in non-immune mice (see text).

A genetic cross between *P. c. chabaudi* CB and AJ was generated (see *Materials and Methods*) and yielded a predicted maximum number of independent recombinant lines of approximately 3,800 (Table 1), as described in *Materials and Methods*. Sporozoites were harvested from the mosquitoes containing the cross and inoculated into non-immune mice as uncloned cross progeny (see *Materials and Methods*). The blood stage parasites of the uncloned cross progeny were subinoculated into mice which had been immunised with one or the other of the parental strains. A non-immune mouse, which was a batch mate of the immunised mice, was inoculated with the same mixture of the uncloned CB x AJ cross progeny. The uncloned cross progeny was allowed to grow in the CB- and AJ-immunised mice and in the non-immune batch mate (Figure 2). The blood stage parasites in the CB- and AJ-immunised mice were harvested and expanded by subinoculation of 10^6^ parasitised Red Blood Cells (pRBC) into groups of four non-immune female CBA mice on days 11 and 12 of infection, respectively. The cross progeny from the non-immune mouse was similarly expanded by subinoculation on day 9 of infection. These parasites were prepared for extraction of parasite genomic DNA for the subsequent analysis, as described in *Materials and Methods*.

**Table 1:** The parameters used to calculate the predicted maximum number of recombinant lines present in the pooled progeny of the genetic cross between strains CB-pyr10 and AJ of *Plasmodium chabaudi chabaudi*.

Proportion of CB and AJ in mixtures used to induce the blood infections	No. of mosquitoes dissected for oocysts (no. infected)	Average no. of oocysts per gut (SEM)	No. of mosquitoes dissected for sporozoites	Predicted no. of oocysts represented	Predicted no. of recombinant lines
1∶1	11 (8)	9.5 (9.26)	190	1,312	2,624
1∶2	16 (7)	4.06 (3.96)	190	337	674
2∶1	18 (13)	3.13 (6.02)	125	283	566
	Total 45 (28)		Total 505	Total 1,932	Total 3,864

The predicted number of such recombinants is calculated as described (see *Materials and Methods*). SEM, standard error of mean.

### Molecular Genetic Analysis of SSPI Selected Uncloned Cross Progeny

The genomic DNA of parasites derived from the CB- and AJ-immune selected cross progeny and from the cross progeny grown in the control non-immunised batch mate was typed with genome-wide quantitative genetic markers produced by Amplified Fragment Length Polymorphism (AFLP) [32] (see *Materials and Methods*). From 97 combinations of selective AFLP primers, 350 polymorphic bands that differentiated CB and AJ strains of *P. c. chabaudi* were obtained. Of these bands, 197 were present only in AJ and 153 were present only in CB. These were used as AFLP markers for the strains AJ and CB, respectively. The Comparative Intensity (CI) (see *Materials and Methods* for a definition) [9] was calculated for each AFLP marker in the CB- and AJ-immune selected cross progeny.

Following AJ-specific immune selection, 24 of the 197 AFLP markers for strain AJ (AJ markers) (12.2% of AJ specific markers) had CIs of less than 50% under AJ-specific immune selection (i.e. intensity reduction of greater than 50% relative to growth of the cross progeny in the non-immune batch mate), listed in Table 2. These AJ markers under SSPI selection were sequenced, as described in *Materials and Methods*. 19 contained a single sequence each, all of which were identified in the *P. c. chabaudi* genome database, at the website http://www.sanger.ac.uk/cgi-bin/blast/submitblast/p_chabaudi. The predicted orthologous loci of these *P. c. chabaudi* contigs were, thereafter, located within the *P. falciparum* genome. Because of the high level of conserved synteny between the genomes of *P. falciparum* and the rodent malaria parasites [33], the chromosomal locations of the *P. falciparum* orthologues of AJ markers could, in turn, be mapped to the equivalent predicted chromosomal positions in the *P. c. chabaudi* genome. The remaining 5 AJ markers under SSPI selection gave unreadable sequence data. Their physical locations in the *P. c. chabaudi* genome could not be determined (Table 2).

**Table 2:** Physical and genetic locations of AFLP markers of *Plasmodium chabaudi chabaudi* strain AJ whose Comparative Intensities (CI) were reduced below 50% in the progeny of the genetic cross between CB-pyr10 and AJ following selection in an AJ-immunised mouse (see text).

Names of *P. c. chabaudi* AJ markers	CIs of the AJ markers in AJ immunised mouse	Physical locations in the *P. falciparum* genome of *P. falciparum* orthologues of the *P. c. chabaudi* AJ markers [46]	*P. falciparum* chromosomes	Chromosome on which AJ marker is predicted to be located in *P. c. chabaudi* by physical mapping	Chromosome on which AJ marker is predicted to be located in *P. c. chabaudi* by genetic mapping	Genetic distances along the *P. c. chabaudi* chromosomes in centi Morgans [34]
AJ TG 03 AA CB	21.7	*pf* 9-378**	9	8	8	22.0
AJ TA 04 AT CB	20.2	*pf* 9-718	9	8	8	43.0
**AJ GT 02 TA CB**	**13.1**	***pf*** ** 9-992**	**9**	**8**	**8**	**43.0**
**AJ AT 01 AG CB**	**10.4**	***pf*** ** 9-1019**	**9**	**8**	**ND**	**ND**
**AJ TT 03 AT CB**	**12.15**	***pf*** ** 9-1113**	**9**	**8**	**8**	**59.4**
**AJ TA 07 TA CB**	**6.01**	***pf*** ** 9-1150**	**9**	**8**	**8**	**59.4**
**AJ TC 01 TG CB**	**1.5**	***pf*** ** 9-1159**	**9**	**8**	**8**	**59.4**
AJ allele of *msp*-1	(0%, 60%)*	*pf* 9-1201	9	8	8	50.8
**AJ AG 05 AG CB**	**1.5**	***pf*** ** 9-1263**	**9**	**8**	**8**	**55.3**
AJ AT 03 AG CB	47.5	*pf* 9-1368	9	8	ND	ND
AJ AC 01 CT CB	31.8	*pf* 11-1084	11	9	9	51.7
AJ AG 01 TC CB	25.9	*pf* 11-1245	11	9	9	63.5
AJ AT 03 TA CB	22.7	*pf* 11-1084	11	9	9	67.2
AJ allele of *ama*-1	(17.3%, 79.6%)*	*pf* 11-1290	11	9	9	71.1
AJ AT 01 GT CB	28.4	ND	ND	ND	7	37.3
AJ TG 01 AT CB	21.09	*pf* 12-1259	12	14	7	77.7
AJ AT 01 TC CB	39.1	*pf* 12-1267	12	14	7	77.7
AJ TA 06 TA CB	23.37	*pf* 12-1290	12	14	7	77.7
AJ TG 01 TC CB	29.0	*pf* 12-1346	12	14	7	77.7
AJ TT 02 TG CB	27.0	*pf* 12-1355	12	14	7	81.4
AJ AG 01 CA CB	43.54	*pf* 4-787	4	7	7	99.6
AJ AG 02 AG CB	27.2	*pf* 14-2756	14	12	ND	ND
AJ TT 01 TC CB	26.88	ND	ND	ND	g2	ND
AJ AG 01 TA CB	40.0	ND	ND	ND	g12	ND
AJ AT 02 TT CB	22.22	ND	ND	ND	g12	ND
AJ TG 02 AT CB	34.6	ND	ND	ND	g12	ND

The six AJ markers with CI of <20% in the AJ-immune selected cross progeny mapped to positions closely linked to the gene encoding the *P. c. chabaudi* merozoite surface protein-1 (MSP-1) are indicated in bold. ND not determined.

* The first and second numbers in brackets represent percentages of parasite DNA carrying the AJ alleles of the indicated gene ( *msp*-1 or *ama*-1), respectively in the AJ-immune selected cross progeny and in the non-immune selected cross progeny, as measured by RTQ-PCR (see text)

** Numbers after ‘*pf*’’ indicate the *Plasmodium falciparum* chromosome number followed by distance along the chromosome in kilo base pairs

Of the 19 AJ markers whose predicted orthologues were successfully identified in the *P. falciparum* genome, one mapped to a region on *P. falciparum* chromosome 4 which is syntenic with a location on *P. c. chabaudi* chromosome 7. Nine other markers were located to a region on *P. falciparum* chromosome 9 which is syntenic with one on *P. c. chabaudi* chromosome 8. Three markers were located to a region on *P. falciparum* chromosome 11 which is syntenic with one on *P. c. chabaudi* chromosome 9. Five additional markers were located to a region on *P. falciparum* chromosome 12 which is syntenic with one on chromosome 14 of *P. c. chabaudi*. A final marker responding to SSPI selection was located to a region on *P. falciparum* chromosome 14 corresponding to one on *P. c. chabaudi* chromosome 12 (Table 2).

**Figure 3:**
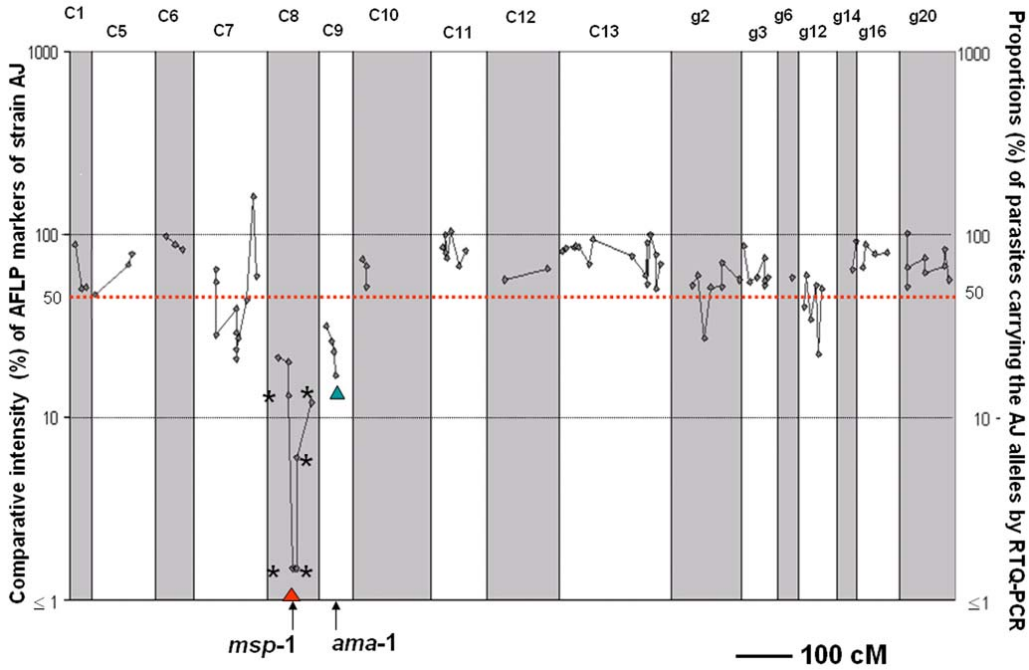
The Comparative Intensities of 92 AFLP markers of *Plasmodium chabaudi chabaudi* strain AJ from the progeny of a genetic cross between *P. c. chabaudi* strains CB-pyr10 and AJ following selection in mice immunised strain AJ (see text). AJ-specific markers (indicated by black diamonds) were located on a *P. c. chabaudi* genetic linkage map, generated from a genetic cross between AS and AJ [34]. Numbers after letter ‘C’ and ‘g’ represent *P. c. chabaudi* chromosome numbers and *P. c. chabaudi* unassigned linkage groups, respectively, in the genetic linkage map [34]. Of the six AJ markers which were most reduced under AJ-specific immune selection (see Table 2), five (indicated by asterisks) could be located to *P. c. chabaudi* chromosome 8, forming a selection valley with the *P. c. chabaudi msp*-1 gene at its lowest point (see text). RTQ-PCR values for the proportions of the AJ-immune selected cross progeny carrying the AJ alleles of the Merozoite Surface Protein-1 (*msp*-1) are indicated by the red triangle and Apical Membrane Antigen-1 (*ama*-1) by the green triangle in the AJ-immune selected cross progeny. The red line indicates Comparative Intensity of 50%.

In addition, 92 of the 197 AJ markers were located onto ten *P. c. chabaudi* chromosomes and six unassigned *P. c. chabaudi* linkage groups of a *P. c. chabaudi* genetic linkage map previously generated from the progeny of a genetic cross between AS and AJ strains [34]. The genetic position of the 92 markers is displayed versus its CI in Figure 3. 21 AJ markers with CIs of <50% following AJ-specific immune selection successfully mapped onto three *P. c. chabaudi* chromosomes and two unassigned *P. c. chabaudi* linkage groups (Table 2). In most cases, the location of the markers by genetic mapping was in agreement with that of the physical mapping. However, five markers that were located to *P .c. chabaudi* chromosome 14 by physical mapping mapped genetically to *P. c. chabaudi* chromosome 7. The reason for this discrepancy is not known and will require further investigation. Four markers whose orthologues could not be identified in *P. falciparum* were genetically located to unassigned *P. c. chabaudi* linkage groups g2 (one marker) and g12 (three markers).

The six AJ markers with the greatest reduction in intensity (CI of below 20%) following the AJ-specific immune selection were all located on *P. c. chabaudi* chromosome 8 by both physical and genetic mapping (represented in bold in Table 2). These AJ markers generally decreased in CI with deceasing physical distance (*P. falciparum*) or genetic distance (*P. c. chabaudi*) from the AJ allele of the *P. c. chabaudi msp*-1 locus (Table 2, Figure 3). The proportion of parasite DNA carrying the AJ allele of *msp*-1 was reduced to undetectable levels (<1%) as measured by strain specific RTQ-PCR in the cross progeny grown under the AJ-specific immune selection compared to its presence at a proportion of 60.0% in the cross progeny grown in the non-immune mouse (Table 2, Figure 3). Thus, the *P. c. chabaudi msp*-1 locus lies at the lowest point that has been detected in the strain specific immunity-selected valley on *P. c. chabaudi* chromosome 8.

Three AJ markers with CI of <50% under AJ-specific immune selection formed a linkage group that contains the gene encoding the *P. c. chabaudi* AMA-1 (Table 2, Figure 3). An *ama*-1 strain specific RTQ-PCR assay for CB and AJ (see *Materials and Methods*) showed that 17.3% of the DNA of parasites in the AJ-immune selected cross progeny carried the AJ allele of *ama*-1 (Table 2), compared to 79.6% carrying the AJ allele of *ama*-1 in the cross progeny grown in the non-immune mouse.

In a reciprocal experiment the uncloned progeny of the cross between *P*. *c. chabaudi* strains CB and AJ were subjected to CB-specific immune selection. The intensities of most of the AFLP markers for strain CB (CB markers) were already very faint in the unselected (control) cross progeny compared to the intensity of the corresponding AFLP bands in the parental strain CB. The general band intensities of the CB markers were below the limit of detection by AFLP in the CB-immune selected cross progeny [35]. Consequently we were unable to achieve a meaningful analysis of the CB-immune selected cross progeny by AFLP. We were, nevertheless, able to investigate the level of CB-specific immune selection against the CB allele at the *msp*-1 and *ama*-1 loci using RTQ-PCR. In DNA from parasites of the CB-immune selected cross progeny the CB allele of *msp*-1 was undetectable (<1%) by RTQ-PCR, compared to 40.0% in the cross progeny grown in the non-immune mouse. At the *ama*-1 locus, 1.8% of the DNA of parasites carried the CB allele of *ama*-1 from cross progeny grown in the CB-immunised mouse, compared to 20.4% in the cross progeny grown in the non-immune mouse.

## Discussion

Linkage Group Selection is a molecular and genetic approach that applies a specific selection pressure to the uncloned recombinant progeny of a genetic cross between two genotypes (strains) of the same species of malaria parasites that differ in their phenotypic responses under the specified selection pressure [36], [37]. Here we have used LGS in an attempt to identify the region(s) in the genome of the rodent malaria parasite *Plasmodium chabaudi chabaudi* which contains the gene(s) for a target of SSPI against the blood stages of this parasite. We have taken two genetically distinct strains of *P. c. chabaudi*, CB and AJ, which induce SSPI with respect to each other in laboratory mice, and crossed them to produce recombinant progeny. We then applied SSPI selection to the uncloned recombinant progeny by growing the cross progeny in mice made immune to one or the other of the two strains of the parasite (strain specific immunised mice). As a reference (LGS control), the same uncloned progeny was also grown in a non-immune batch mate of the strain specific immunised mice. The cross progeny grown in the CB- or AJ-immunised mouse, or the non-immune batch mate, was screened for the presence and intensity of quantifiable genome-wide AFLP markers distinguishing CB and AJ.

Following growth of the cross progeny in the AJ-immunised mouse, a small proportion of markers of the immunising strain (AJ markers) was judged to be significantly reduced (CI of <50%) relative to the progeny grown in the non-immune mouse. These markers were located in the genome by either physical or genetic mapping, or both, as described in *Results*. Several AJ markers that were located to a region on *P. c. chabaudi* chromosome 8 formed the deepest identified selection valley in this analysis (Figure 3). Within this selection valley, the two AJ markers under the strongest AJ-specific immune selection spanned a predicted ∼120 kb region (based upon the predicted physical locations of the orthologues of these two markers in the *P. falciparum* genome) containing a gene for the *P. c. chabaudi* MSP-1, a principle candidate for a target antigen of SSPI. RTQ-PCR analysis confirmed that the uncloned cross progeny carrying the AJ allele of the *msp*-1 gene was virtually eliminated after selection in the AJ-immunised mouse. The reciprocal result was obtained after selection of the cross progeny in a CB-immunised mouse. In this case parasites carrying the CB allele of the *P. c. chabaudi msp*-1 locus were virtually eliminated. These results demonstrate that parasites carrying AJ or CB alleles in the region of the *msp*-1 locus were under very strong selection in AJ- or CB-immunised mice, respectively. A previous LGS analysis of SSPI in the progeny of a genetic cross between strains AS-pyr1 and CB of *P. c. chabaudi* also found that the region containing the *msp*-1 locus was under the strongest detected strain specific protective immune selection for this combination of strains [9]. Together, these results strongly support the view that the region on *P. c. chabaudi* chromosome 8 around the *msp*-1 locus contains a gene or genes encoding a major target antigen or antigens of SSPI.

In addition to those AJ markers associated with the selection valley around *msp*-1, several other AJ markers had CIs of less than 50% after selection in the AJ-immunised mouse (Table 2, Figure 3), suggesting the possible presence of other targets of SSPI.

Several of these more weakly reduced AJ markers formed linkage groups having possible association with selection valleys. One such group, containing seven affected AJ markers, was located on *P. c. chabaudi* chromosome 7. This group spanned 62.3 centiMorgan (cM) by genetic mapping along chromosome 7, but there was uncertainty as to the location of these markers in the genome by physical mapping (see Table 2). Two other such markers were located on *P. c. chabaudi* unassigned linkage groups 2 and 12 (Table 2). The reductions in intensity of all these markers after selection in the AJ-immunised mouse were, however, much less than those associated with the *msp*-1 locus, suggesting relatively small contributions toward SSPI. No obvious candidate gene for a target antigen of SSPI has been identified within these genomic regions.

Three of the relatively weakly affected AJ markers were located on *P. c. chabaudi* chromosome 9 and were linked to the gene for AMA-1 (Table 2), another candidate target of SSPI [31]. The results suggest that AMA-1, or the product of a gene closely linked to that for AMA-1, may be a target of SSPI, but one that is less strongly affected than the target(s) associated with the gene for MSP-1.

As already noted we had previously conducted LGS analysis of SSPI with a combination of *P. c. chabaudi* strains, AS-pyr1 and CB [9]. However, in contrast to the situation in the present cross between AJ and CB, the AS-pyr1 and CB strains have DNA sequences which are identical at the *ama*-1 locus for the extracellular domain of the AMA-1 protein (Cheesman and Carter, unpublished data). It is, therefore, highly unlikely that evidence of the involvement of AMA-1 in SSPI could have been found in the AS-pyr1 and CB cross, nor was it [9].

There is a possibility that there are other regions of the *P. c. chabaudi* genome that may be under SSPI selection pressure but have not been detected, as no AFLP markers have been mapped on *P. c. chabaudi* chromosomes 2, 3, 4 and 14 and only a few on chromosomes 10 and 12 (Figure 3). Nevertheless, quite large chromosomally unassigned linkage groups (see Figure 3) were identified. These almost certainly represent groups of linked markers on all, or most, of the missing chromosomes [34]. Since no clear selection valley has been located on any of these unassigned linkage groups, it is unlikely that our analysis failed to identify any other major SSPI-associated selection valleys that might exist.

LGS analyses of SSPI have, however, been conducted on the progeny of two different genetic crosses, one between AJ and CB-pyr10 (in this present study) and the other between AS-pyr1 and CB (the parental clone of CB-pyr10) [9]. Both analyses have revealed prominent selection valleys at the same location on *P. c. chabaudi* chromosome 8. In both cases, the base of the SSPI-selected valley covered a region within ∼60 kb on either side of the *msp*-1 locus. We conclude, therefore, that the *P. c. chabaudi msp*-1 locus, or a gene very closely linked to it, dominated the SSPI selection in both of these combinations of *P. c. chabaudi* strains. Although our work has not yet proved that the *P. c. chabaudi* MSP-1 protein is itself a target of SSPI in malaria, such a result would be consistent with the literature that indicates the involvement of MSP-1 in the strain specific protective immune response in the rodent malarias. Thus, on passive transfer into non-immune mice, monoclonal antibody (mAb) NIMP23, which had been raised against the MSP-1 protein of *P. c. chabaudi* strain AS, inhibited the growth of blood stages of the homologous strain (AS) [29], but had no effect on a heterologous challenge infection with strain CB of *P. c. chabaudi* [30]. Following these studies, McKean and colleagues were able to identify an epitope to which mAb NIMP23 bound and which was located at the C-terminus of *P. c. chabaudi* MSP-1 [38]. This part of MSP-1 was shown to be genetically distinct between strains AS and CB ([39], Cheesman and Carter, unpublished data). The MSP-1 sequence in the same region is also different between strains AJ and CB ([39], Cheesman and Carter, unpublished data), that are the two parental strains used in the present LGS analysis of SSPI. However, between strains AS and AJ, two strains which show virtually no SSPI with respect to each other (Carter and Martinelli, unpublished data), there is no difference in the amino acid sequence of MSP-1 in this region [39]. The sequence polymorphisms identified at the C-terminus of protein sequence within this region of MSP-1 in *P. c. chabaudi* are, therefore, consistent with the involvement of MSP-1 in SSPI in this parasite.

In conclusion, SSPI to blood stage malarial infections has been observed in malarias of rodents [7], [9], [10]. Using LGS analyses, we have identified a region on *P. c. chabaudi* chromosome 8 which is under strong SSPI selection and which contains the gene for *P. c. chabaudi* MSP-1. No other region in the *P. c. chabaudi* genome was found to contain a similarly strong target of SSPI.

## Materials and Methods

### Parasites, Laboratory Mice and Mosquitoes

Two cloned strains of the rodent malaria species *P. c. chabaudi* denoted CB-pyr10 and AJ were used in these experiments. CB-pyr10 is a pyrimethamine resistant cloned line derived through pyrimethamine selection from CB (a pyrimethamine sensitive line), originally cloned from isolate CB [40]. In this study, CB-pyr10 will be referred to as CB for simplicity. AJ is a pyrimethamine sensitive line, cloned from isolate AJ [18]. Both cloned lines are known to be genetically distinct from each other [18]. AJ and CB were isolated from wild thicket rats, *Thamnomys rutilans*, captured in the same locality in the Central African Republic in 1969 and 1970, respectively [17], [41]. Maintenance of the parasite lines is described in detail elsewhere [10].

Females of inbred laboratory mouse strains, CBA/Ca and C57BL/6J, were used, aged 5 to 6 weeks old on first infection. These mouse strains will be referred here to as CBA and C57. Mosquitoes were from a laboratory-bred colony of *Anopheles stephensi*. Mice and mosquitoes were maintained as previously described [42], [43]. All animal work in this study was carried out in accordance with the Animals (Scientific Procedures) Act 1986 UK.

### Induction and characterisation of SSPI between cloned strains CB and AJ of *P. c. chabaudi*

Immunity was induced in groups of CBA mice by two consecutive rounds of infection with *P. c. chabaudi* followed by mefloquine cure with slight modifications from the method previously described [10]. In brief, each experimental mouse was inoculated intra-peritoneally (i.p.) with 5×10^5^ pRBC of either strain, CB or AJ, of *P. c. chabaudi*. Blood stage malarial infections were terminated by four consecutive daily doses of 20 mg/kg mouse body weight of mefloquine in 0.1 ml Dimethyl Sulfoxide, by oral gavage. Mefloquine treatment started before parasitaemias exceeded 50% but after they had passed 20% of infected red blood cells. The blood stage parasites were monitored daily by microscopic examination of thin blood smears stained with Giemsa's stain. Following each mefloquine treatment all mice remained blood smear negative. Twenty-six days after the last day of the first mefloquine treatment, mice were given a second i.p. infection with 5×10^6^ pRBC of the *P. c. chabaudi* strain homologous to the first immunising one. Infections were grown for 5 days and drug cure treated as before.

Sixteen weeks after the second mefloquine treatment, two mice previously immunised by blood stage parasite infection with either CB or AJ from each group were randomly chosen to test for the presence of SSPI. Each mouse was challenged with a mixture of 5×10^6^ pRBC containing an equal proportion of blood stage parasites, CB and AJ. An equivalent inoculum from the same mixture was inoculated into a non-immune mouse, which was a batch mate of the immunised mice. The resulting infections were examined daily by microscopic analysis. A 20 µl sample of tail blood in physiological citrate saline (0.85% (w/v) sodium chloride, 1.5% (w/v) tri sodium citrate dihydrate, in distilled water, adjusted to pH 7.2 with hydrochloric acid) was collected from each mouse 5–8 days post infection, so as to accurately measure proportions of parasites of each strain (CB and AJ) using strain specific RTQ-PCR for CB and AJ alleles of the *msp*-1 gene [10], [44]. Tail blood samples were spun at 5,000 rpm for 3 minutes. The red blood cell pellet was washed with 200 µl phosphate buffered saline (Sigma) and stored at −70°C prior to DNA extraction using a High Pure PCR template preparation kit (Roche Diagnostics). RTQ-PCR analysis was performed as previously describe by the method of Cheesman et al., [10], [44].

### Preparation of a Genetic Cross between Cloned Strains CB and AJ of *P. c. chabaudi*

Single strain infections with CB and AJ were individually grown in donor C57 mice. Parasites of the two strains were harvested from the donors and accurately mixed to produce three inocula in proportions of 1∶1, 1∶2 and 2∶1 of CB and AJ, respectively. The mixtures were inoculated i.p. at 10^6^ pRBC per mouse into three groups of C57 mice (four mice each). The parasitaemias from the mixed strain infections were followed microscopically on thin tail blood smears stained with Giemsa's stain. Six days post inoculation of the parasites, when parasitaemias were ∼20% and the presence of gametocytes of both sexes was confirmed microscopically, mice from each mixed strain infection group were anaesthetised and placed on a mosquito cage, each containing ∼250 female mosquitoes. Mosquitoes were aged 5–7 days post emergence from pupae. The mosquitoes were allowed to feed on the mice for 20 minutes without interruption. The mice were thereafter humanely killed before recovery from the anaesthesia. Eight days after the blood meal, samples of mosquitoes from each cage were dissected and examined for the presence of oocysts on their midguts. 11 mosquitoes from those fed on the 1∶1 CB:AJ mixture had a mean of 9.5 oocysts per gut (Standard Error of Mean, SEM = 9.26); 16 mosquitoes from those fed on the 1:2 CB:AJ mixture had a mean of 4.06 oocysts per gut (SEM = 3.96); 18 mosquitoes from those fed on the 2:1 CB:AJ mixture had a mean of 3.13 oocysts per gut (SEM = 6.02).

Sixteen days after the blood meal, when sporozoites were present in the mosquito salivary glands, the mosquitoes were allowed to feed on 6 non-immune CBA female mice in order to naturally transmit sporozoites of the progeny of the CB×AJ genetic crosses. All surviving mosquitoes of these three batches were then dissected for sporozoites from the salivary glands (190 mosquitoes from the 1∶1 CB:AJ mixture, 190 mosquitoes from the 1∶2 CB:AJ mixture and 125 mosquitoes from the 2∶1 CB:AJ mixture). The glands were gently crushed in 0.2–0.4 ml volumes of 1∶1 Foetal Bovine Serum (GIBCO BRL): Ringer's solution (2.7 mM Potassium Chloride, 1.8 mM Calcium Chloride, 154 mM Sodium Chloride) and injected i.p. into 8 non-immune CBA mice. All 14 mice, naturally infected or inoculated with sporozoites dissected from salivary glands of the mosquitoes, became infected. The blood stage parasites representing the progeny of the genetic cross were harvested on day 9 post infection (mean parasitaemia = 15%, min = 0.6% and max 61%) and pooled prior to inoculation of mice previously immunised with parasites of either one or the other of the parental strains CB or AJ for SSPI selection, or else into a non-immune batch mate. The remaining pooled blood was frozen as stabilates and stored in liquid nitrogen.

From the data on the number of oocysts per mosquito and the number of mosquitoes dissected for inoculation of sporozoites (given above), it is possible to estimate the likely maximum numbers of recombinant lines that a genetic cross could have generated. On an assumption that gametes of the two parasite strains are generated in equal numbers in the mixed strain infection used to make a genetic cross, and that fertilisations occur randomly between them, half of all fertilisations will generate hybrid zygotes between the gametes of the two parental strains of parasite, while the second half will derive equally from the self fertilisations representing one or other of the two parental strains. Each hybrid zygote undergoes meiosis and produces four recombinant progeny lines. Thus, on average, for every four oocysts in such a genetic cross, there will be two parental and two hybrid oocysts, yielding a total of eight recombinants. Overall, therefore, there are, on average, two recombinant lines for every oocyst present. Using this logic we were able to estimate the likely maximum number of recombinant lines present in the pooled cross progeny (Table 1).

### SSPI Selection of Uncloned Cross Progeny for Linkage Group Selection Analysis

The blood stage parasites from a total of 14 non-immune CBA mice (six and eight mice from the natural transmission and the sporozoite-induced infection, respectively) were harvested in physiological saline and pooled to produce the blood inoculum containing the uncloned CB×AJ cross progeny. One mouse from the batches immunised with blood stage parasite of either CB or AJ and previously tested for SSPI to these strains was inoculated i.p. with 5×10^6^ pRBC of the uncloned cross progeny. At time of infection with the cross progeny, the immunised mice were twenty weeks after the last day of the second mefloquine treatment (see above). The blood stage parasites from the cross progeny grown in these mice which had been previously immunised with CB or AJ, were designated “CB-immune selected cross progeny” or “AJ-immune selected cross progeny”, respectively. An equal number of the cross progeny designated “non-immune selected cross progeny” was passaged into a non-immune batch mate of the immunised mice. The resulting infections were followed by microscopic examination of thin blood smears stained with Giemsa's stain. The uncloned cross progeny was grown in the CB- and AJ-immunised mice for 11 and 12 days, respectively. In the non-immune batch mate, the cross progeny was allowed to grow for 9 days. To grow parasites in sufficient quantities to prepare DNA for subsequent analysis, blood was, thereupon, collected, and 10^6^ pRBC from each mouse were inoculated into four non-immune mice. The parasites were grown in these mice to parasitaemias of ∼35–50% before harvesting and preparing them for DNA extraction, as previously described by Grech et al., [32].

### Quantitative Measurement of Strain Specific Amplified Fragment Length Polymorphism (AFLP) Markers

To locate regions of the *P. c. chabaudi* genome affected by the SSPI selection, we generated ^33^P-radiolabelled AFLP markers from genomic DNA of the CB- and AJ-immune selected cross progeny, the non-immune selected cross progeny and the two parental cloned strains (CB and AJ) by the method of Grech et al., [32]. AFLP products were resolved on 6% denaturing polyacrylamide gel electrophoresis and visualised using a Phosphoscreen (Amersham) and an X-ray film (Kodak). The intensities of AFLP bands were quantitatively measured using ImageQuant™ software version 1.2 Build 039 (Molecular Dynamics), as described by Martinelli et al., [35]. For the purpose of determining the proportions of the parasite population carrying a specific allele for CB or AJ represented by individual AFLP markers in the immune and non-immune selected materials, we calculated a “Relative Intensity Index (RII)”. This is defined as the intensity of the strain specific AFLP marker band in the mixture divided by the intensity of a designated non-polymorphic band in the same mixed parasite sample, divided by the equivalent ratio of the two relevant bands (strain specific AFLP and non-polymorphic band) measured in a sample of the pure parental strain [35]. The RII of parasites bearing specific AFLP markers for CB and AJ in the immune selected cross progeny (RII_i_) were, thereafter, quantitatively compared to the RII of the same markers derived from the non-immune selected cross (RII_ni_). These proportions were, then, used to calculate the “Comparative Intensity (CI)”- a measurement of proportional changes of strain specific AFLP markers between the immune and non-immune selected cross progeny. The CI is defined by the RII of a strain specific AFLP marker of the immune-selected cross progeny (RII_i_) divided by the RII of the relevant marker of the non-immune selected cross progeny (RII_ni_), and expressed as a percentage, i.e., CI = (RII_i_/RII_ni_)×100 [9]. Specific AFLP markers of the immunising parasite strain whose CI values were less than 50% were considered to be under SSPI selection [9].

### Physical and Genetic mapping of AFLP markers under SSPI selection

Strain specific AFLP markers that had CI reduced below 50% following the strain specific immune selection were identified and characterised by the methods of Hunt et al., [45] and Martinelli et al., [9]. The physical location of AFLP markers under SSPI selection was conducted as follows. Individual AFLP markers were excised and eluted from the polyacrylamide gel into distilled water to obtain DNA fragments of each marker. The extracts were PCR amplified using the same pairs of AFLP primers and conditions that were used to generate the original AFLP markers [32]. PCR products were directly sequenced on both strands using the same AFLP primers. The sequencing reactions used ABI BigDye Terminator Chemistry on ABI3700 sequencing machine, according to manufacturer's instructions. For PCR products of AFLP markers less than 100 bp, DNA sequences were obtained by cloning with a TOPO TA cloning kit™ (Invitrogen). Plasmid DNA from each transformed colony was extracted using a QIAprep Spin Mini Prep kit (QIAGEN) and sequenced as described above. DNA sequences obtained from the forward and reverse primers were visualised using an EditView ABI automated DNA sequence viewer software (Perkin Elmer ABI) and assembled manually to generate a single contiguous sequence. The sequences of the specific AFLP primers were eliminated from the assembled DNA sequence prior to location of the AFLP marker onto the genome databases by BLAST search.

Eight-fold coverage contigs of the genome of *P. c. chabaudi* cloned strain AS, http://www.sanger.ac.uk/cgi-bin/blast/submitblast/p_chabaudi, were searched using the BLASTN (DNA vs. DNA) option with sequences derived from the AFLP markers. Genomic contigs of *P. c. chabaudi* which contained DNA sequences corresponding to the relevant AFLP marker with lowest Probability score (a cut-off of 90% sequence identity) were obtained. The chromosomal positions of these contigs have not been mapped physically in the *P. c. chabaudi* genome. Sequences of whole *P. c. chabaudi* contigs were, thereafter, used to locate orthologous loci in the *Plasmodium falciparum* (cloned strain 3D7) genome database [46] (http://www.ncbi.nlm.nih.gov/sutils/blast_table.cgi?taxid = Protozoa&database), using the blastn option. The genomic locations of the *P. falciparum* orthologues were, in turn, used for physical mapping to chromosomal locations in the genome of *P. c. chabaudi* through the conserved genetic synteny between the human malaria parasite *P. falciparum* and rodent malaria parasites [33].

In addition to physical mapping, the strain specific AFLP markers were genetically located on a *P. c. chabaudi* genetic linkage map which was constructed with reference to the progeny of a genetic cross between *P. c. chabaudi* strains AS and AJ (see [34] for the method).

### RTQ-PCR of CB and AJ alleles of *P. c. chabaudi msp*-1 and *ama*-1

RTQ-PCR analysis was performed on a LightCycler instrument (Roche Diagnostics) as described by Cheesman et al., [10], [44] for allele specific amplification assays to measure in the mixtures of parasites the proportions of DNA of the CB or AJ allele of the *msp*-1 or *ama*-1 gene. The assays have been standardized for accurate quantification of the CB or AJ alleles of *msp*-1 or *ama*-1 of the blood stage parasites, using artificial mixtures with known proportions of cloned strains CB and AJ as previously described by Cheesman et al., [44]. DNA samples of (i) the blood stage mixed strain infections and (ii) the uncloned CB x AJ cross progeny grown in the strain specific immunised and non-immune mice were prepared as described in previous sections. DNA samples of the pure parental strains CB and AJ were 10-fold serially diluted in the range 66-0.0066 ng and used as quantification standards to construct a DNA concentration calibration curve. Oligonucleotide primers were designed to selectively amplify a strain specific region of the *msp*-1 or *ama*-1 gene (Cheesman and Carter, unpublished data). The *msp*-1 specific primers used in the assay were: the CB forward and reverse primers: 5′-AGTTGTTCCTGTGGCAG-3′ and 5′-CTGTTACAACCCAAACC-3′; and the AJ forward and reverse primers: 5′-ACTGAAGCAACAACACCAGC-3′ and 5′-GTTGTTGATGCACTTGCGGGTTC-3′. RTQ-PCR reactions for the CB and AJ alleles of *msp*-1 were set up, as previously described [10]. The *ama*-1 specific primers used in the assay were: the CB forward and reverse primer 5′-AGGTTTCATT**A**TTAACACGAG-3′ and 5′-GATTACTTTTGTC ATAAACAGCG-3′; and the AJ forward and reverse primer 5′ CTAAATCATT**C**TTAGA CCC-3′ and 5′-GGCATAATTTTTATATTCTG-3′. The positions at which nucleotides of the CB and AJ *ama*-1 alleles mismatched are shown in bold. RTQ-PCR reactions for quantification of the CB and AJ alleles of *ama*-1 were performed in a 10 µl volume of standard buffer containing 4 µM of forward and reverse primers in 3 and 2.5 mM MgCl_2_, respectively. The PCR conditions used were as follows: An initial “Hot Start” at 95°C for 600 s followed by 40 cycles of 95°C with a 0 s hold, cooling at 20°C/s to 58°C with a 7 s hold for CB or cooling at 20°C/s to 56°C with a 7 s hold for AJ, reheating at 20°C/s to 72°C with a 15 s hold for CB or 10 s hold for AJ and finally heating at 20°C/s to 72°C with a 0 s hold. Melting curve data for each PCR run was produced, as follows: Heating at 20°C/s to 95°C with 0 s hold, cooling at 20°C/s to 65°C with 30 s hold and reheating at 0.2°C/s to 95°C in a continuous data acquisition mode. The final step of RTQ-PCR was at 20°C/s to 40°C with a 60 s hold. Data obtained from each LightCycler run were checked to ensure that the correct strain specific melting peak and no other non-specific amplicon was produced. The DNA concentrations measured on the LightCycler were converted into relative proportions of the AJ and CB alleles for *msp*-1 or *ama*-1 within each sample analysed.
